# Endogenous testosterone density as ratio of endogenous testosterone levels on prostate volume predicts tumor upgrading in low-risk prostate cancer

**DOI:** 10.1007/s11255-021-03008-0

**Published:** 2021-10-22

**Authors:** Antonio Benito Porcaro, Sebastian Gallina, Alberto Bianchi, Clara Cerrato, Alessandro Tafuri, Riccardo Rizzetto, Nelia Amigoni, Rossella Orlando, Emanuele Serafin, Alessandra Gozzo, Filippo Migliorini, Stefano Zecchini Antoniolli, Vincenzo Lacola, Vincenzo De Marco, Matteo Brunelli, Maria Angela Cerruto, Salvatore Siracusano, Alessandro Antonelli

**Affiliations:** 1 Department of Urology, University of Verona, Azienda Ospedaliera Universitaria Integrata, Piazzale Stefani 1, 37126 Verona, Italy; 2 Department of Pathology, University of Verona, Azienda Ospedaliera Universitaria Integrata, Verona, Italy

**Keywords:** Prostate cancer, Low-risk prostate cancer, Radical prostatectomy, Tumor upgrading, Endogenous testosterone (ET), ET density (ETD), Prostate-specific antigen (PSA), PSA density (PSAD), Percentage of biopsy positive cores (BPC), BPC density (BPCD)

## Abstract

**Objectives:**

To evaluate preoperative endogenous testosterone (ET) density (ETD), defined as the ratio of ET on prostate volume, and tumor upgrading risk in low-risk prostate cancer (PCa).

**Materials and methods:**

From November 2014 to December 2019, 172 low-risk patients had ET (nmol/L) measured. ETD, prostate-specific antigen density (PSAD) and the ratio of percentage of biopsy positive cores (BPC) to prostate volume (PV), defined as BPC density (BPCD), were evaluated. Associations with tumor upgrading in the surgical specimen were assessed by statistical methods.

**Results:**

Overall, 121 patients (70.3%) had tumor upgrading, which was predicted by BPCD (odds ratio, OR = 4.640; 95% CI 1.903–11.316; *p* = 0.001; overall accuracy: 70.3%). On multivariate analysis, tumor upgrading and clinical density factors related to each other for BPCD being predicted by ETD (regression coefficient, *b* = 0.032; 95% CI 0.021–0.043; *p* < 0.0001), PSAD (*b* = 1.962; 95% CI 1.067–2.586; *p* < 0.0001) and tumor upgrading (*b* = 0.259; 95% CI 0.112–0.406; *p* = 0.001). According to the model, as BPCD increased, ETD and PSAD increased, but the increase was higher for upgraded cases who showed either higher tumor load but significantly lower mean levels of either ET or PSA.

**Conclusions:**

As ETD increased, higher tumor loads were assessed; however, in upgraded patients, lower ET was also detected. ETD might stratify low-risk disease for tumor upgrading features.

## Introduction

Prostate cancer (PCa) is a health priority for being the second most commonly cancer detected in the aging male [1, 2]. Once the disease is diagnosed, stratification of patients into risk groups is pivotal for the patient management [1, 2]. The low-risk category is a heterogenous set of patients in whom early detection may be associated with overdiagnosis and, as such, overtreatment [1, 2]. Treatment approaches include several options that vary from active surveillance (AS) and watchful waiting (WW), which are no-active treatments, to radical prostatectomy (RP) and radiotherapy (RT). Furthermore, other therapeutic options include cryotherapy and high-intensity focused ultrasound, but are recommended within clinical trial settings [3, 4]. Pelvic lymph node dissection (PLND) is performed when the risk of cancer invasion varies from 2% to more than 5%, according to international guidelines [1, 2]. However, reclassification and/or biochemical persistence as well as progression may occur in the low-risk category for upgrading and upstaging issues; furthermore, side effects related to active treatments are also drawbacks for indolent disease [1, 2]. So far, more clinical parameters are needed to stratify low-risk patients according to cancer aggressive features; as a result, appropriate managements may be decided to improve quality of life features [1, 2].

Endogenous testosterone (ET) is the most important circulating androgen impacting on prostate growing disorders [5]. Prostate-specific antigen (PSA), prostate volume (PV) and PSA density (PSAD), which is the ratio of circulating PSA to PV are also important factors for evaluating prostate diseases [1, 5]. Since ET is decreasing during aging, it has been indicated as a potential risk factor for PCa together with other metabolic features such as diet, obesity, and metabolic syndrome [1, 2, 5]. Because of the close hormonal dependency of the prostate gland on ET levels, it has been suggested that ET levels may associate with different levels of PCa aggressivity; however, the issue remains controversial for controlled studies are missing [6–8]. Recently, we have shown that ET density (ETD) together with PSAD associated with the risk of high tumor load in the surgical specimen of low-risk PCa patients [9]. In the present study, we wanted to test the hypothesis of potential associations of ETD with cancer aggressive features including tumor upgrading in a larger cohort of low-risk PCa patients.

## Materials and methods

### Features of the study population

The study was approved by Institutional Review Board. Informed consent was obtained by all subjects. Data were collected prospectively but evaluated retrospectively. In a period ranging from November 2014 to December 2019, 805 consecutive PCa patients who were not under androgen blockade had ET (nmol/L) measured at our lab before surgery and the test was performed at least 1 month after biopsies between 8.00 and 8.30 a.m. by radioimmunoassay. PSA (ng/mL), age (years), body mass index (BMI; kg/m^2^), PV (mL) and percentage of biopsy positive cores (BPC, % defined as the number of positive cores on the number of total cores taken) were evaluated in each case. PV was calculated by transrectal ultrasound (TRUS) standard method. Biopsies performed elsewhere were assessed for number of cores taken, tumor grade and PV measured by TRUS. In our Institution, the 14-core trans-perineal technique was used [10]. In each case, we also adjusted BPC, PSA and ET as densities related to PV; as such BPC density (BPCD, %/mL); PSAD (ng/mL^2^) and ETD [nmol/(L mL)] were calculated as the ratio of BPC, PSA and ET to PV, respectively. Clinical staging was assessed by the TNM system, accordingly [1, 2]. Finally, patients were classified into risk classes [1, 2].

The decision to perform surgery in low-risk PCa patients was taken according to the time-related guidelines indication and after patients counseling illustrating therapeutic options as active surveillance, radical prostatectomy, and RT even considering patients intention. Also, the presence of factors predicting tumor upgrading and upstaging coming from our previous experience was considered [11, 12]. The decision to perform PLND was based on clinical factors indicating increased risk of tumor upgrading and lymph node invasion (LNI) in the surgical specimen [13, 14]. Surgery, which was delivered by robot-assisted radical prostatectomy (RARP) or open radical prostatectomy (ORP), was performed by experienced surgeons. Nodal packets were submitted in separate packages according to a standard anatomical template including bilateral external iliac, obturator, Marcille’s common iliac, and Cloquet’s nodal stations [15, 16]

Removed prostates were placed into formalin, weighted and evaluated by the dedicated pathologist who graded the tumors according to the International Society of Urological Pathology (ISUP) system [1, 2]. Tumor quantitation was assessed as tumor load (TL), defined as the percentage of prostate volume invaded by cancer in the surgical specimen [1, 2]. Surgical margins were stated positive when cancer invaded the inked surface of the specimen. Removed lymph nodes were assessed for number and cancer invasion. Surgical specimens were then staged by the TNM system, accordingly [1, 2].

### Statistical methods

The study wanted to test the hypothesis of associations between ETD and tumor upgrading features in low-risk PCa category defined as PSA < 10 and GS ≤ 6 (ISUP 1) and cT1c-2a, according to the European Association of Urology (EAU) guidelines. Continuous variables were measured for means (standard deviation, SD) and medians (interquartile range, IQR). Categorical factors were assessed for frequencies (percentages). Associations of clinical factors with tumor upgrading were evaluated by correlation analysis that also assessed relations between factors (univariate analysis). Correlations with pathological features were also evaluated. The association with the risk of tumor upgrading was evaluated by the logistic regression model, which was also evaluated for accuracy fit. Associations of tumor upgrading with clinical density factors including ETD, PSAD and BPCD was finally evaluated by the linear regression model (multivariate analysis). Figures were derived from logistic and linear regression models performed (univariate and multivariate analysis). Further details are reported in the description of each figure. The software used to run the analysis was IBM-SPSS version 26. All tests were two-sided with *p* < 0.05 considered to indicate statistical significance.

## Results

### Demographics and associations with tumor upgrading

**Fig. 1:**
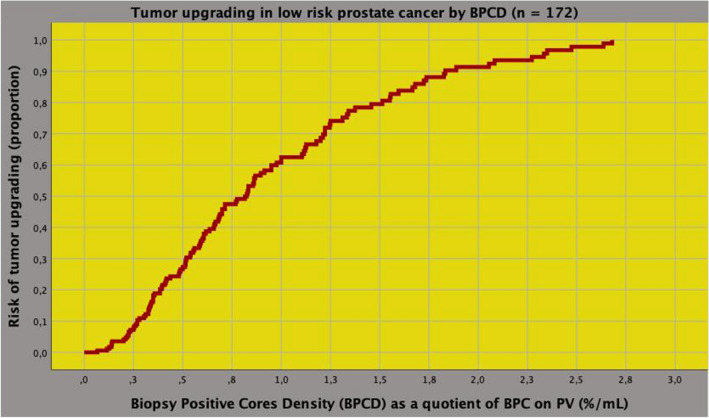
Risk curve of tumor upgrading in low-risk prostate cancer (PCa) including 172 cases. The density of percentage biopsy positive cores (BPCD; %), as the ratio of BPC on prostate volume (%/mL), was a strong predictor of the risk, which increased as density of BPC increased (odds ratio, OR = 4.640; 95% CI 1.903–11.316; *p* = 0.001). The model showed a good fit for overall accuracy being 70.3%

Demographics of the low-risk population including 172 cases is reported in Table 1. RARP was the most frequent approach, which was performed in 156 cases (90.7%). Cancer invasion extended beyond the prostate in 16 subjects (9.3%) and surgical margins resulted positive in 39 cases (22.7%). Pelvic lymph node dissection was performed in 77 (44.8%) patients of whom 3 (3.9%) had cancer invasion. The distribution of the ISUP system included grade I in 51 cases (29.7%), grade II in 73 subjects (42.4%), grade III in 38 patients (22.1%), grade IV in 7 cases (4.1%) and grade V in 3 subjects (1.7%). Overall, 121 patients (70.3%) had upgraded tumors, which correlated either to load (Pearson’s correlation coefficient, *r* = 0.216; *p* = 0.004) and extracapsular extension of cancer (*r* = 0.165; *p* = 0.030). Of all clinical factors, only BPC (*r* = 0.257; *p* = 0.001) and their density (*r* = 0.273; *p* < 0.0001) associated with the risk of tumor upgrading, which was stronger for the BPCD (odds ratio, OR = 4.640; 95% CI 1.903–11.316; *p* = 0.001) when compared to the former (OR = 1.044; 95% CI 1.017–1.072; *p* = 0.001). As shown in Fig. 1, the risk of tumor upgrading increased as density of BPC increased; furthermore, the model showed a good fit for overall accuracy being 70.3%.

**Table 1:** Baseline characteristics of patients with low-risk prostate cancer stratified by tumor upgrading in the surgical specimen

	Population	Tumor upgrading in the surgical specimen		Univariate analysis	
		No	Yes		
*N* (%)	172	51 (29.7)	121 (70.3)	*r* (*)	*p* value
**Clinical factors**					
Age (years)				0.063	0.414
Mean (SD)	64.5 (6.4)	63.8 (6.6)	64.7 (6.3)		
Median (IQR)	65 (60–70)	65 (59–69)	65 (61–70)		
Body mass index; BMI (kg/m^2^)				− 0.022	0.779
Mean (SD)	26.3 (3.3)	26.4 (3.4)	26.2 (3.2)		
Median (IQR)	25.9 (24.1–28)	25.8 (24.2–28.7)	26 (24.0–27.9)		
Prostate-specific antigen; PSA (ng/mL)				− 0.061	0.427
Mean (SD)	6 (2.1)	6.2 (2)	5.9 (2.1)		
Median (IQR)	6.1 (4.5–7.5)	6.2 (4.7–7.8)	6 (4.5–7.4)		
PSA density [PSAD; ng/(mL mL)]				0.053	0.493
Mean (SD)	0.15 (0.08)	0.14 (0.08)	0.15 (0.08)		
Median (IQR)	0.14 (0.10–0.19)	0.12 (0.09–0.19)	0.13 (0.10–0.19)		
Endogenous testosterone; ET (ng/dL)				0.012	0.427
Mean (SD)	408.9 (146.4)	406.1 (135.6)	410 (151.2)		
Median (IQR)	388. 3 (299.1–492.2)	375 (315–469.8)	397.7 (295–490.5)		
ET density [ETD; ng/(dL mL)]				0.120	0.117
Mean (SD)	10.8 (6.4)	9.6 (4.7)	11.2 (6.9)		
Median (IQR)	9.2 (6.5–13.6)	8.3 (6.3–12.5)	9.3 (6.6–14.2)		
Biopsy positive cores; BPC (%)				0.257 (*)	**0.001**
Mean (SD)	27.9 (16.7)	21.3 (11.7)	30.7 (17.7)		
Median (IQR)	25 (14–33.7)	19 (14–29)	27 (17–42)		
BPC density (BPCD; %/mL)				0.273 (**)	** < 0.0001**
Mean (SD)	0.74 (0.55)	0.51 (0.32)	0.84 (0.60)		
Median (IQR)	0.58 (0.33–1.00)	0.42 (0.26–0.70)	0.66 (0.37–1.18)		
Prostate volume; PV (mL)				− 10.8	0.157
Mean (SD)	44.6 (17.8)	47.6 (18.6)	43.3 (17.4)		
Median (IQR)	42 (31.6–53.7)	44 (36–55)	40.8 (30–52.5)		
Tumor stage (cT); *n* (%)				− 0.073	0.338
1c	117 (68)	32 (62.7)	85 (70.2)		
2a	55 (32)	19 (37.3)	36 (29.8)		
ASA; *n* (%)				0.137	0.172
I	20 (11.6)	9 (17.6)	11 (9.1)		
II	140 (81.4)	40 (78.4)	100 (82.6)		
III	12 (7)	2 (3.9)	10 (8.3)		
Pathological factors					
Prostate weight; PW (g)				− 0.024	0.754
Mean (SD)	57.7 (20.5)	58.5 (20.4)	57.4 (20.6)		
Median (IQR)	45 (55–70)	55 (46–72)	55 (40.6–79)		
Tumor load; TL (%)				**0.216**	**0.004**
Mean (SD)	16.5 (11.9)	12.5 (10.9)	18.1 (11.9)		
Median (IQR)	10 (15–30)	8 (5–15)	15 (10–25)		
Pathological tumor stage (pT)				**0.165**	**0.030**
pT2	156 (90.7)	50 (98)	106 (87.6)		
pT3a	10 (5.8)	1 (2)	9 (7.4)		
pT3b	6 (3.5)	0 (0)	6 (5)		
Positive surgical margins (PSM)				0.108	0.157
No	133 (77.3)	43 (84.3)	90 (74.4)		
Yes	39 (22.7)	8 (15.7)	31 (25.6)		
Pathological nodal stage (pN)				0.111	0.336
pN0	74 (43)	18 (35.3)	56 (46.3)		
pN1	3 (1.7)	0 (0)	3 (2.5)		
pNx	95 (55.3)	33 (64.7)	62 (51.2)		

*SD* standard deviation, *IQR* interquartile range, *r *Pearson's correlation coefficient, *OR* odds ratio, *CI* confidence interval; (*), OR = 1.044 (95% CI 1.017–1.072; *p* = 0.001; (**), OR 4.640 (95% CI 1.903–11.316; *p* = 0.001)

### Correlations between clinical factors not directly related to tumor upgrading

Analysis of clinical factors not including BPC with relative densities is described in Table 2. Interestingly, ET inversely correlated to PSA (*r* = − 0.211; *p* = 0.005), so that PSA levels increased as ET decreased and vice versa, and to BMI (*r* = − 0.217; *p* = 0.004). Moreover, PSA directly correlated to BMI (*r* = 0.212; *p* = 0.005) and PV (*r* = 0.187; *p* = 0.014), but inversely to cT (*r* = − 0.170; *p* = 0.026). Adjusting for volumes of the prostate, ETD and PSAD were strongly correlated to each other (*r* = 0.398; *p* < 0.0001) such that as ETD increased PSAD increased. Further details are illustrated in the Table 2.

**Table 2:** Correlation analysis of clinical variables not including BPC in low-risk prostate cancer (*n* = 172 cases)

	Age	BMI	ASA	PSA	PSAD	ET	ETD	PV	cT
Age		0.045	**0.250**	0.134	0.063	− 0.034	− 0.086	0.062	0.081
		0.554	**0.001**	0.079	0.414	0.654	0.261	0.419	0.288
BMI			**0.208**	**0.212**	− 0.006	− **0.217**	− **0.237**	**0.225**	− 0.115
			**0.006**	**0.005**	0.933	**0.004**	**0.002**	**0.003**	0.132
ASA				0.021	0.008	− 0.028	− 0.023	0.004	0.045
				0.782	0.916	0.720	0.768	0.956	0.555
PSA					**0.562**	− **0.211**	− **0.237**	**0.187**	− **0.170**
					** < 0**.**0001**	**0.005**	**0.002**	**0.014**	**0.026**
PSAD						− 0.086	**0.398**	− **0.582**	− **0.169**
						0.262	** < 0**.**0001**	** < 0**.**0001**	**0.027**
ET							**0.636**	− 0.052	0.018
							** < 0**.**0001**	0.497	0.815
ETD								**0.641**	− 0.008
								** < 0**.**001**	0.919
PV									0.030
									0.700

See Table 1.

### Associations of ETD with tumor load measured by BPCD predicting tumor upgrading risk

As BPCD is defined as the ratio of BPC on prostate volume (PV) it is influenced by PV: patients presenting with same BPC values show different densities according to PV, with BPCD increasing as PV decreases and vice versa.

**Fig. 2:**
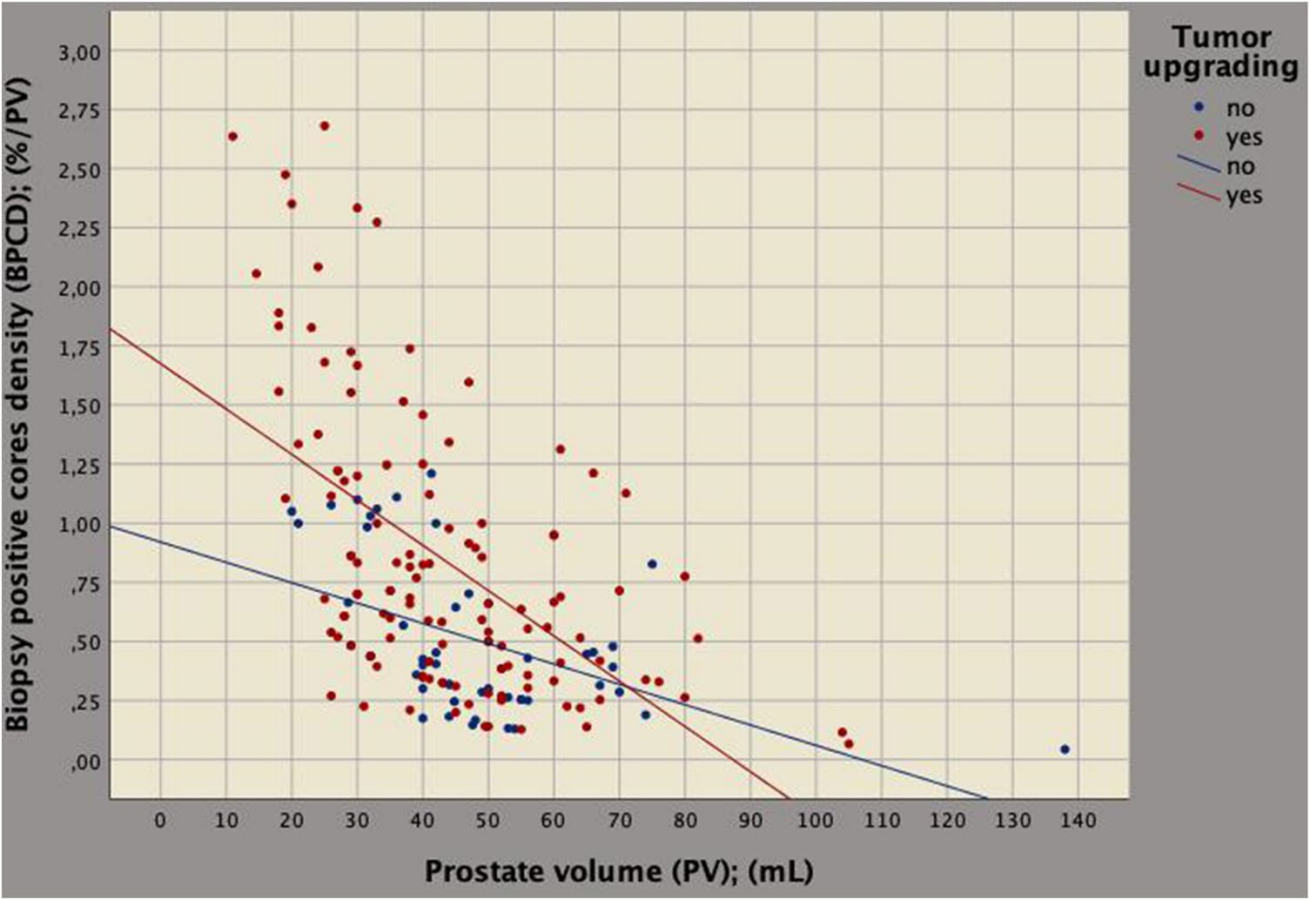
Biplot diagram showing the relation between prostate volume (PV; mL) versus percentage of biopsy positive cores density (BPCD; %) on PV (BPCD; %/mL). As PV decreased, BPCD increased, but the increase was significantly higher for upgraded patients. See results for further details

In the surgical specimen, BPCD strongly correlated to tumor load (*r* = 0.246; *p* = 0.001); so far, as BPCD increased, a greater quantity of tumor load was detected in the removed prostates, as well. Table 3 compares associations of BPC and BPCD with clinical and upgrading factors, as well. On univariate analysis, BPCD strongly correlated to PV (*r* =− 0.530; *p* < 0.0001); so far, BPCD increased as prostate volumes decreased (inverse association) with upgraded patients showing higher densities (Fig. 2); furthermore, BPCD correlated to ETD (*r* = 0.509; *p* < 0.0001), PSAD (*r* = 0.446; *p* < 0.0001) and tumor upgrading (*r* = 0.273; *p* < 0.0001), as well. BPCD increased as ETD and PSAD increased.

**Table 3:** Clinical factors associated with percentage of biopsy positive cores that predicts tumor upgrading in the surgical specimen

Statistics	Percentage of biopsy positive cores (BPC)		Density of percentage of biopsy positive cores (BPCD)			
	Univariate analysis		Univariate analysis		Multivariate analysis	
	*r*	*p* value	*r*	*p* value	*b* (95%CI)	*p* value
Age	− 0.027	0.722	− 0.065	0.993		
BMI	0.095	0.214	− 0.041	0.591		
ASA	0.005	0.952	− 0.018	0.811		
PSA	− 0.010	0.898	− 0.095	0.213		
PSAD	0.051	0.506	**0.446**	** < 0**.**0001**	**1**.**962 (1**.**067–2**.**586)**	** < 0**.**0001**
ET	− 0.057	0.455	0.016	0.838		
ETD	0.033	0.666	**0.509**	** < 0**.**0001**	**0**.**032 (0**.**021–0**.**043)**	** < 0**.**0001**
PV	− 0.050	0.514	− **0.530**	** < 0**.**0001**		
cT	− 0.132	0.083	− 0.090	0.239		
BPC			**0.738**	** < 0**.**0001**		
Tumor upgrading	0.257	0.001	**0.273**	** < 0**.**0001**	**0**.**259 (0**.**112–0**.**406)**	**0.001**

*r* Pearson's correlation coefficient, *b* linear regression coefficients, *CI* confidence interval

**Fig. 3:**
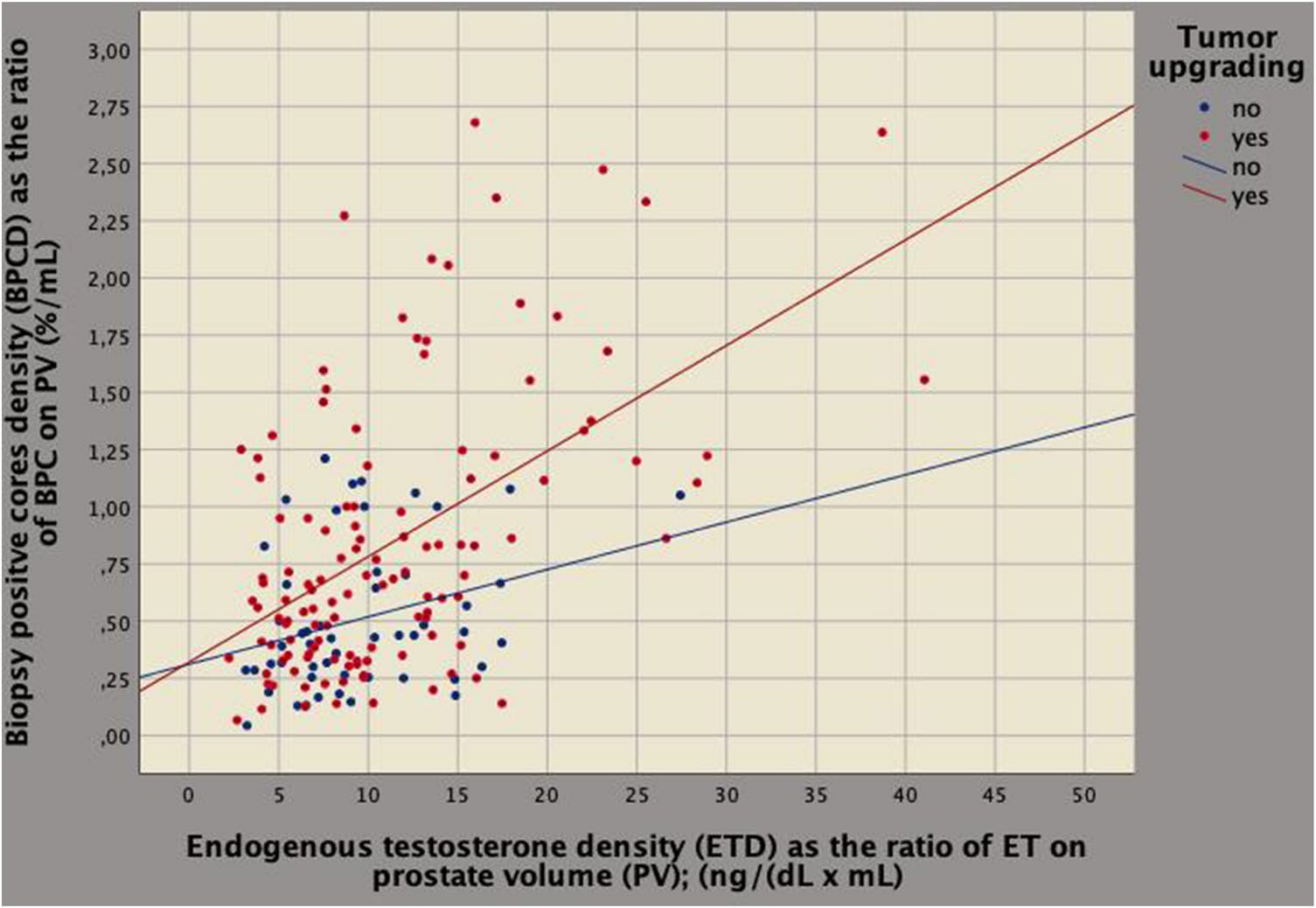
Biplot diagram showing relationships between endogenous testosterone density (ETD), as the ratio of endogenous testosterone on prostate volume [ng/(dL mL)] versus density of biopsy positive cores (BPCD, %/ml). As BPCD increased, ETD also increased, but the amount was higher for upgraded cases compared with the control group

**Fig. 4:**
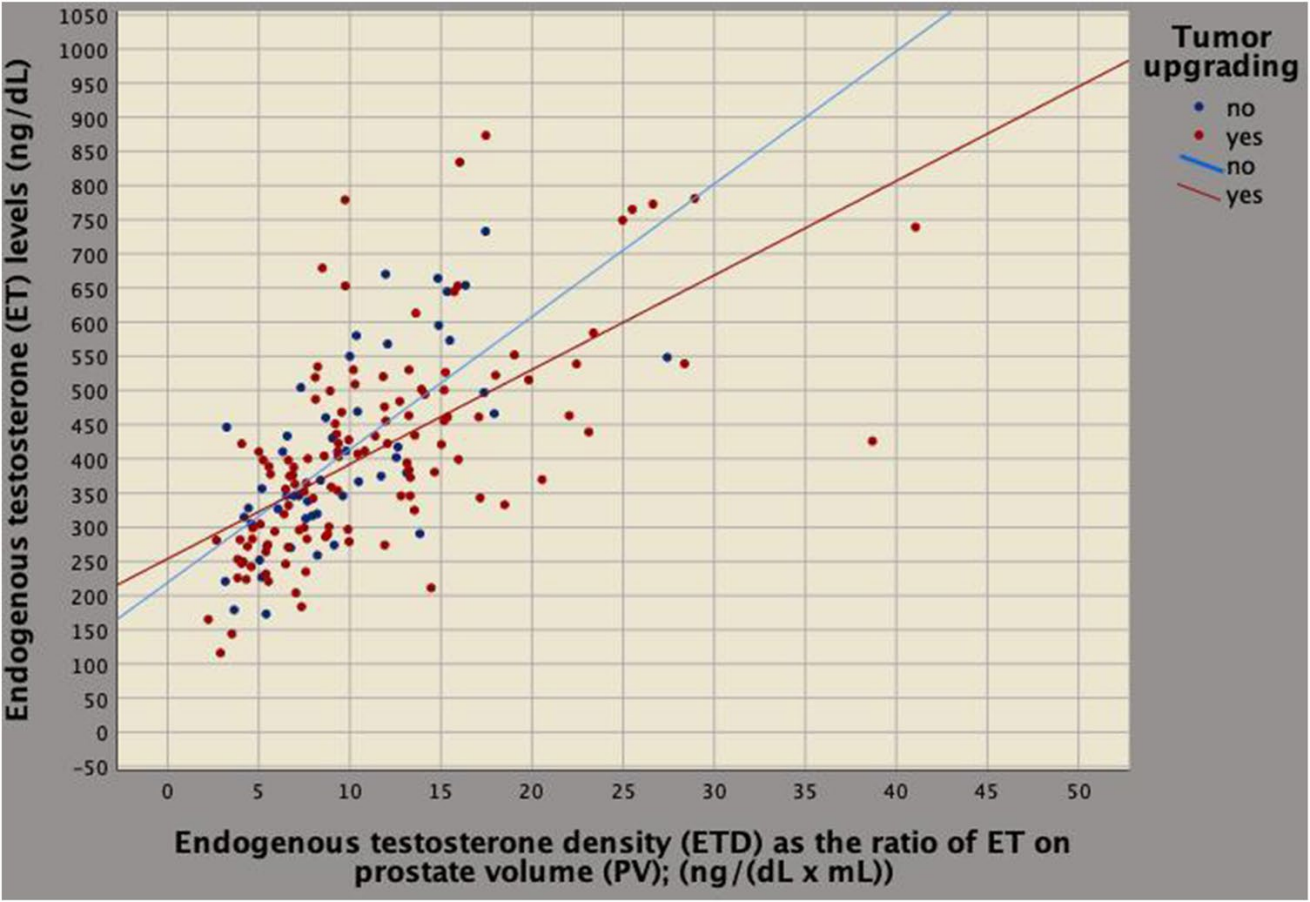
Biplot diagram showing relationships between endogenous testosterone density (ETD), as the ratio of endogenous testosterone (ET) to prostate volume [ng/(dL mL)] versus ET (ng/dL). As ETD increased, ET increased also; however, upgraded patients had significantly lower mean ET levels when compared to the control group

**Fig. 5:**
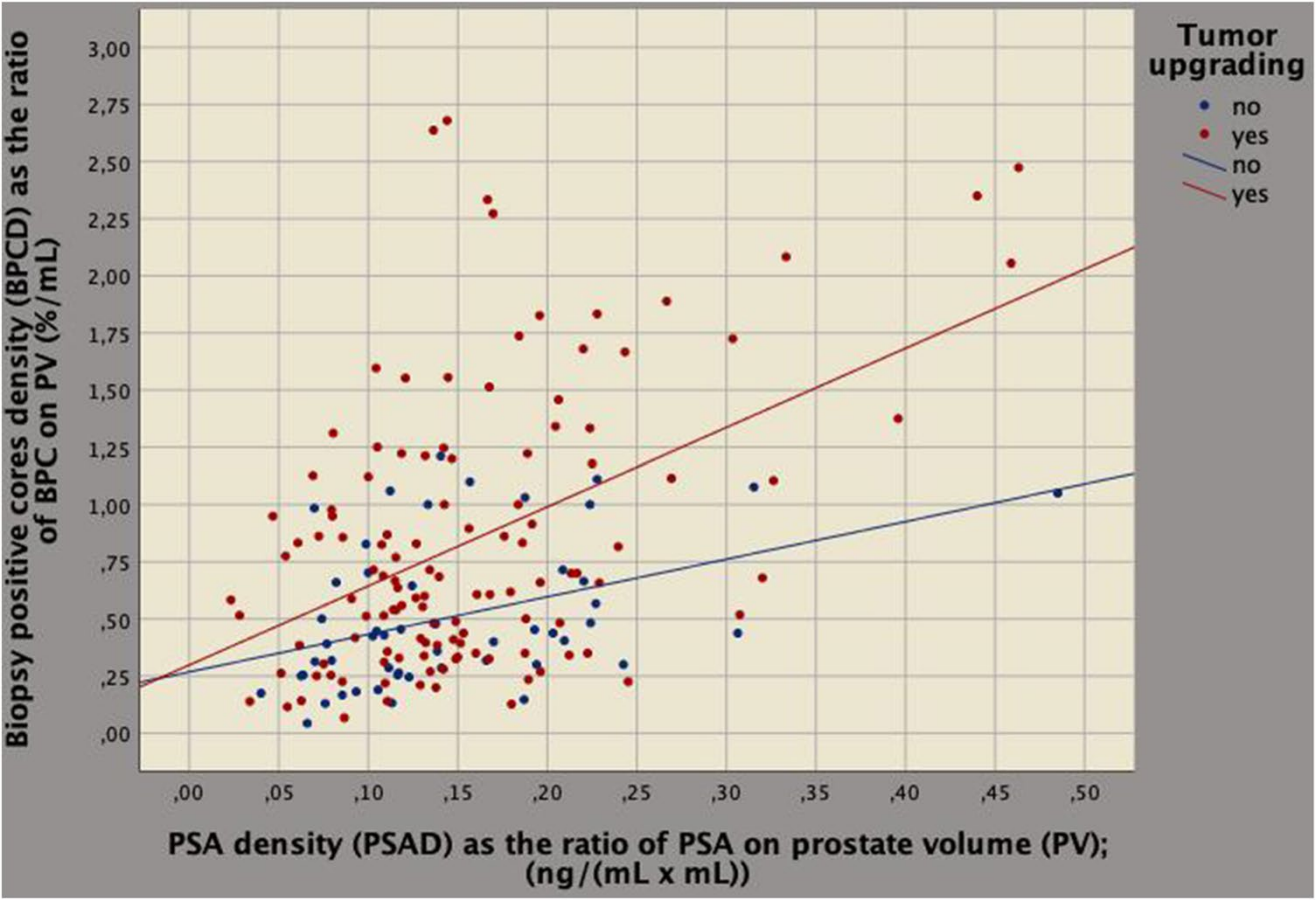
Prostate-specific antigen (PSA) density (PSAD), as the ratio of PSA on prostate volume [ng/(dL mL)] versus density of biopsy positive cores (BPCD; %/mL). As BPCD increased, PSAD raised up; however, upgraded patients showed significantly higher mean PSAD levels

**Fig. 6:**
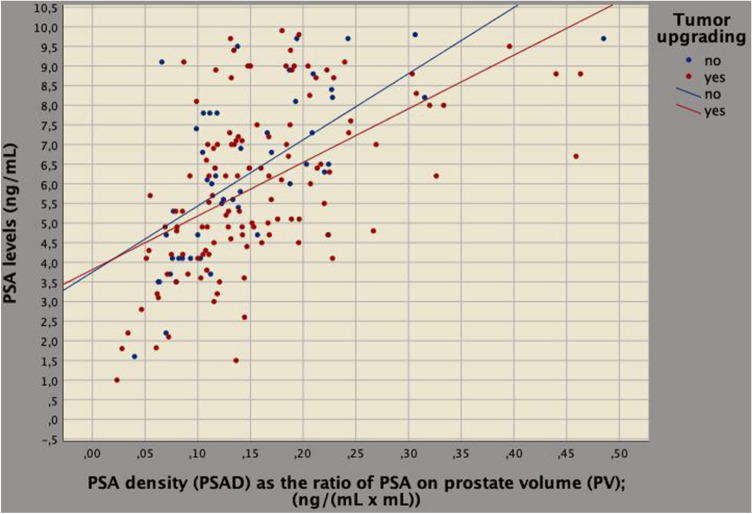
Prostate-specific antigen (PSA) density (PSAD), as the ratio of PSA to prostate volume [ng/(dL mL)] versus PSA (ng/mL). As PSAD measurements increased, endogenous PSA levels raised up; however, upgraded patients showed significantly lower mean levels of PSA

On multivariate analysis, tumor upgrading and clinical density factors related to each other for BPCD being predicted by ETD (regression coefficient, *b* = 0.032; 95% CI 0.021–0.043; *p* < 0.0001), PSAD (*b* = 1.962; 95% CI 1.067–2.586; *p* < 0.0001) and tumor upgrading (*b* = 0.259; 95% CI 0.112–0.406; *p* = 0.001). According to the model, as BPCD increased also ETD increased (Fig. 3). Tumor load (represented by BPCD) was higher for upgraded cases; however, among upgrading patients, those having the same ETD values showed lower mean levels of ET compared to patients who did not upgraded, as shown in Fig. 4. As a result, aggressive tumors are associated with lower ET levels. Furthermore, as BPCD increased, PSAD increased (Fig. 5), but the increase was higher in upgraded patients who had higher tumor load in the biopsy cores, but significantly lower PSA mean levels (Fig. 6).

## Discussion

Low-risk PCa is a heterogenous category of patients for being under assessed for either disease severity or extension. As a result, tumor upgrading in the surgical specimen is an issue for rates ranging from 43 to 63.8% [17, 18]. Recently, a multicenter observational study from the UK has shown that upgrading rates were higher for the low-risk category when compared to either the intermediate and high-risk categories (55.7% vs 19.1% and vs 24.3%, respectively) [19].

Although low-risk subjects under AS represent a highly selected cohort, disease reclassification with tumor upgrading is even a more serious drawback for the delayed active treatment of occult aggressive disease. This particular set of patients show upgrading rates that vary from 30% up to 50%, as well; a retrospective study found out that the risk of tumor upgrading was 49.3% in low-risk patients undergoing surgery when AS inclusion criteria were considered [20, 21]. Here we considered low-risk PCa patients who underwent radical prostatectomy at our tertiary center after counseling considering the presence of clinical factors predicting tumor upgrading, upstaging, and LNI in the pathological specimens as PSA, serum TT levels, and positive core number as well as patients’ intention [11–14]. We found a rate of tumor upgrading similar to data reported in the recent literature.

More predictors of tumor upgrading are needed to stratify (PCa patients belonging to the low-risk category). Literature reports of large cohorts have shown that tumor upgrading may be predicted by several factors including PSA, ET, number of positive cores, percentage of cancer involvement in each core, PV, PSAD and BMI; furthermore, such factors have been included in nomograms, as well [17–20, 22, 23]. Accordingly, low-risk patients elected to AS are more likely to be upgraded when presenting with small prostates, increased PSAD and BMI as well as with low ET levels, but high tumor load at prostate biopsy is the most important factor; however, these studies did not specifically investigate the low-risk cohort, but population sets including all risk classes having biopsy Gleason score 6 and PSA within 10 ng/mL; as result, these findings do not specifically apply for the low-risk category [20]. Recently, two studies investigating on the role of multiparametric magnetic resonance imaging (mpMRI) in cases with biopsy Gleason score 6 have shown that mpMRI might have a role in predicting tumor upgrading in the surgical specimen; however, these studies had limitations for investigating all risk classes and not specifically the low-risk category [24, 25]. So far, the effective role of ET on tumor upgrading in low-risk disease is biased by methodological limits of these studies [13, 26, 27]. Actually, few studies have investigated on factors associated with the risk of tumor upgrading in low-risk patients treated with RP. These trials have shown that patients presenting with low-risk disease are more likely to be upgraded for factors related to physical status (age, BMI), cancer specific features (number of positive cores and percentage of cancer involving each core), prostate glandular factors (PV, PSAD), hematological features (neutrophil, platelets and eosinophil to lymphocyte ratio) and genetic factors (expression levels of specific microRNAs); furthermore, ETD has been shown to associate with the risk of high tumor load in the surgical specimen; thus, predicting an unfavorable pathological outcome [9, 28–34]. This study demonstrated the importance of classifying low-risk PCa patients according to the density of BPC, which is strongly associated with occult aggressive disease in the surgical specimen. As BPCD raised, the risk of tumor upgrading increased; as a result, larger prostates were less exposed to such risk. BPCD, as a parameter, gives a better idea of the “quantity of tumor” that resides in the prostate than BPC, which simply describes the percentage of positive cores.

Moreover, our study also demonstrated that ET and PSA, when related to volume of the gland, associated with aggressive tumor load, as expressed by BPCD, which predicted either load or upgrading of the tumor in the surgical specimen. Upgraded patients were more likely to have increased ETD and PSAD, which either associated with raised BPCD measurements. Upgraded patients had significantly lower mean levels of ET and PSA, as well. So far, aggressive PCa in low-risk subjects undergoing surgery associated with lower mean levels of either ET or PSA. This means that low-risk subjects who had same BPC densities were more likely to be upgraded for either low ET and PSA levels. Taken together, all these results, which represent a novelty, have implications for either explanations or managing low-risk PCa.

The results of our study showed associations with cancer biology in low-risk disease; as such, these findings need explanations. As reported, upgraded tumors associated with high tumor load on biopsy specimens, which showed significantly lower mean levels of either ET or PSA. These findings might be explained by the evidence that ET levels are decreasing in the aging male for the impairment of Leydig cells that produce the hormone [35]. As a result, prostate epithelial cells need appropriate levels of ET to evolve up to well-differentiated androgen-dependent cells [35]. So far, prostate malignant disorders when associated with low intraprostatic ET levels for enlarged prostates, epithelial cells undergo cancer induction because intraglandular diffusion of testosterone is insufficient to provide differentiation up to androgen-dependent cells [5, 9, 35]. The dynamics of ET levels as well as of their prostate densities may associate with metabolic disorders and increasing BMI so that all these changes impact by promoting cancer induction and progression in an environment where epithelial cells are poorly differentiated for not being exposed to appropriate levels of testosterone; furthermore, lower PSA amounts are produced by the specific cells [5, 9, 35–37].

Our study has several limits. First, it was retrospective and thus suffers these kinds of biases.

Second, ET was measured only once and thus this may be not sufficient for an appropriate evaluation of its dynamics. Third, we did not explore the hypothalamic–pituitary–gonadal axis as well as estradiol levels, which correlate with ET and BMI dynamics. Fourth, prostate volumes were not all evaluated in our institution and this may cause potential biases in measurement variations. Fifth, biopsies performed outside our institution were not reviewed by the dedicated pathologist; however, we have already shown that no significant differences of upgrading rates have been detected between biopsies performed at our Institution versus elsewhere [30, 31]. Finally, histological features like lymphovascular and perineural invasion was not considered.

Our study has also several strengths. First, it was a single center investigation and thus the low-risk population was homogenous for evaluated features. Second, all ET measurements were assessed in the morning between 8.00 and 8.30 to avoid afternoon variations when significantly lower levels are detected [38]. Third, all volumes of the prostate were measured by TRUS for volume variations are biased to a lesser degree than suprapubic methods. Finally, all specimens were evaluated by our dedicated pathologist.

Our study has also implications in clinical practice. Low-risk patients may be stratified according to standard factors as well as according to densities related to ET, BPC and PSA, as well. Low-risk patients are likely to be upgraded as BPCD increase; however, subjects presenting with the same BPCD, are more likely to be upgraded for lower mean levels of ET and PSA, as well. As such, densities of ET, BPC and PSA all relate to high tumor load, which is a feature of tumor upgrading and upstaging.

## Conclusions

As ETD increased, higher tumor loads were assessed; however, in upgraded patients, lower ET were also detected. ETD might stratify low-risk disease for tumor upgrading features.
